# Epithelial CEBPD activates fibronectin and enhances macrophage adhesion in renal ischemia-reperfusion injury

**DOI:** 10.1038/s41420-024-02082-4

**Published:** 2024-07-18

**Authors:** Shen-Shin Chang, Chao-Chun Cheng, Ying-Ren Chen, Feng-Wei Chen, Ya-Min Cheng, Ju-Ming Wang

**Affiliations:** 1 Division of Transplantation, Department of Surgery, National Chung Kung University Hospital, College of Medicine, National Cheng Kung University, Tainan, 701 Taiwan; 2 Institute of Basic Medical Sciences, College of Medicine, National Cheng Kung University, Tainan, 701 Taiwan; 3 Department of Pathology, National Cheng Kung University Hospital, College of Medicine, National Cheng Kung University, Tainan, 701 Taiwan; 4 Department of Obstetrics and Gynecology, National Cheng Kung University Hospital, College of Medicine, National Cheng Kung University, Tainan, 701 Taiwan; 5 Department of Obstetrics and Gynecology, Kuo General Hospital, Tainan, 700 Taiwan; 6 Department of Biotechnology and Bioindustry Sciences, College of Bioscience and Biotechnology, National Cheng Kung University, Tainan, 701 Taiwan; 7 Graduate Institute of Medical Sciences, College of Medicine, Taipei Medical University, Taipei, 110 Taiwan; 8 Graduate Institute of Medicine, College of Medicine, Kaohsiung Medical University, Kaohsiung, 807 Taiwan

**Keywords:** Extracellular matrix, Genetic engineering, Apoptosis

## Abstract

Ischemia-reperfusion injury (IRI) is a cause of acute kidney injury in patients after renal transplantation and leads to high morbidity and mortality. Damaged kidney resident cells release cytokines and chemokines, which rapidly recruit leukocytes. Fibronectin (FN-1) contributes to immune cell migration, adhesion and growth in inflamed tissues. CCAAT/enhancer-binding protein delta is responsive to inflammatory cytokines and stresses and plays functional roles in cell motility, extracellular matrix production and immune responses. We found that the expression of CCAAT/enhancer-binding protein delta was increased in renal epithelial cells in IRI mice compared with sham mice. Following IRI, the colocalization of FN-1 with the macrophage marker F4/80 was increased in renal injury model wild-type mice but was significantly attenuated in *Cebpd*-deficient mice. Inactivation of CEBPD can repress hypoxia-induced FN-1 expression in HK-2 cells. Moreover, the inactivation of CEBPD and FN-1 also reduces macrophage accumulation in HK-2 cells. These findings suggest that the involvement of CEBPD in macrophage accumulation through the activation of FN-1 expression and the inhibition of CEBPD can protect against renal IRI.

## Introduction

Ischemia-reperfusion injury (IRI) is a detrimental condition for which physicians must develop strategies to attenuate cell damage and preserve organ function. Renal IRI, which is an unavoidable consequence of renal transplantation due to blood flow occlusion, often leads to acute kidney injury in transplant recipients, thus contributing substantially to morbidity and mortality [1, 2]. Inflammatory responses affect the quality and survival of renal allografts [3]. Although the complexity of the pathophysiology and detailed mechanisms of IRI continue to be recognized, strategies for preventing the consequences of IRI remain challenging. Therefore, the dissection of critical factors related to IRI is important for improving renal transplant outcomes.

IRI results in an inflammatory response during kidney procurement, with underlying factors that involve hypoxia, leukocyte extravasation and cell death. Recent studies have demonstrated that injured renal tubular epithelial cells are responsive to external and internal stresses and contribute to the upregulation of proinflammatory gene expression [4]. In addition, several tissue IRI animal models have been used to study macrophage-epithelial interactions (MEIs) [5–9]. In response to damaged or affected cells, such as epithelial or dendritic cells, macrophage infiltration is responsive to the production of inflammatory mediators and plays a crucial role in both innate and adaptive immune responses in the kidneys following ischemia. However, the details of the regulation and relationships among protein molecules and cellular phenomena in renal MEI during IRI remain less well characterized.

The adhesion of leukocytes to the vascular endothelium is a hallmark of the inflammatory response. The original leukocyte adhesion cascade is initiated through integrin-mediated arrest, selectin-dependent rolling, and chemokine-triggered activation. Leukocyte arrest is instigated by chemokines or similar chemoattractants, leading to the binding of leukocyte integrins to intercellular adhesion molecule 1 (ICAM1) and vascular cell adhesion molecule 1 (VCAM1) expressed on endothelial cells, thereby facilitating their adhesion [10]. Additionally, several studies have indicated that fibronectin (FN-1), a crucial component of the extracellular matrix (ECM), plays a significant role in the adhesion of various cell types. The synthesis or absence of FN-1 can influence cell adhesion [11–14].

The transcription factor CCAAT/enhancer-binding protein delta (CEBPD) belongs to the CCAAT/enhancer-binding protein family. It plays functional roles in cell differentiation, motility, growth arrest, cell death, metabolism, ECM production and immune responses [15–18]. In addition to highly conserved protein sequences, the critical motifs involved in transcriptional regulation are almost identical between the human *CEBPD* and mouse *Cebpd* genes [19]. Inflammation, including macrophage accumulation and activation marked by the release of cytokines, has been implicated in initiating renal IRI. The role of the ECM in modulating immune cell migration into inflamed tissues has been previously neglected. Recent studies have suggested that ECM fragments are upregulated during inflammation and consequently perpetuate inflammatory responses, including the regulation of macrophage adhesion [20–22]. The expression and regulation of ECM components, such as FN-1 and laminin, modulate macrophage adhesion and play important roles in inflammation [23, 24]. A recent study showed that CEBPD contributes to the transcriptional activation of the *FN1* gene in glioblastoma under hypoxia [25]. However, whether the regulation of *FN1* gene transcription is responsive to CEBPD in epithelial cells and its consequent effect on IRI tissue remain open questions.

## Results

### Cebpd transcripts are significantly upregulated in kidney transplantation rats and a mouse model of kidney ischemia-reperfusion

**Fig. 1:**
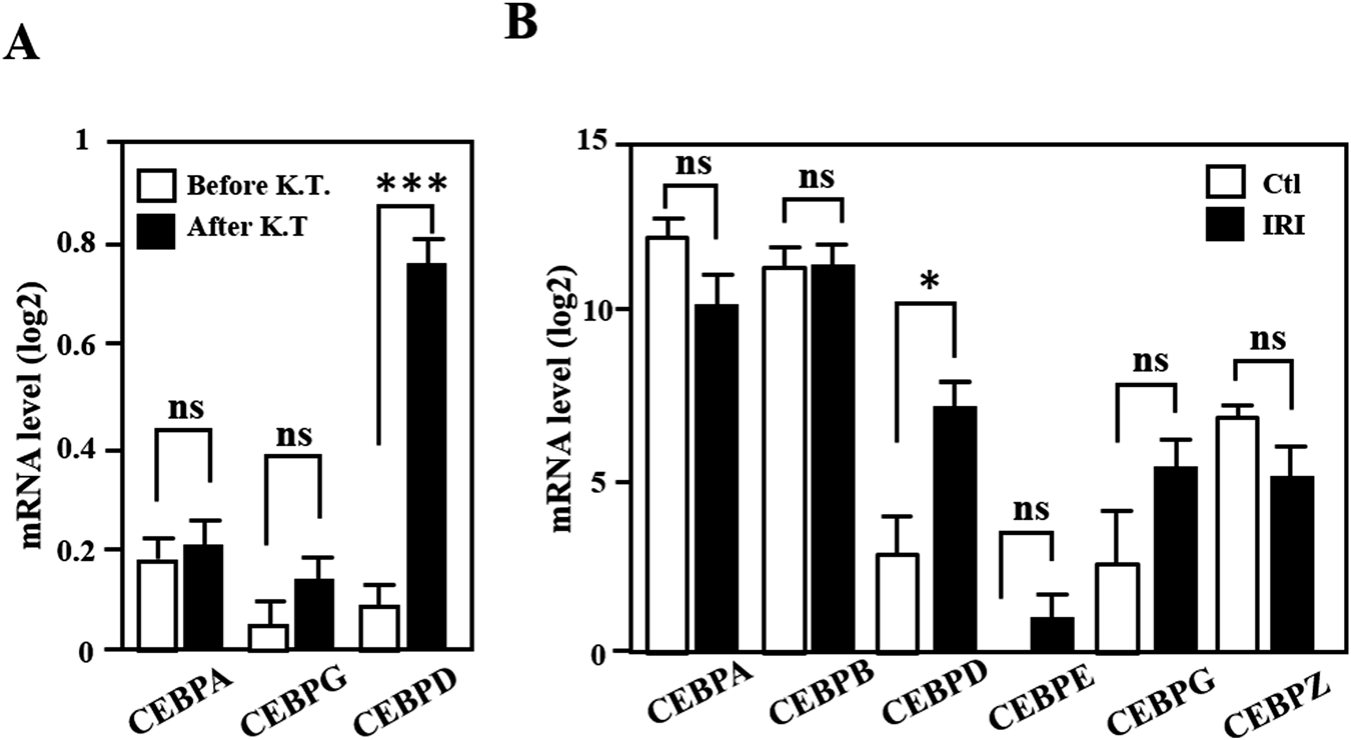
*CEBPD* is significantly upregulated in a kidney transplant rat model and a kidney ischemia-reperfusion mouse model. **A** The relative mRNAs of *Cebpd, Cebpa* and *Cebpg* were extracted from the GEO (GSE5104) database. Comparing the before and after kidney transplantation (K. T.), the *Cebpd*, but not *Cebpa* and *Cebpg*, mRNA was highly increased. **B** The relative mRNAs of the indicated C/EBP family members from the GEO (GSE182793) database. Comparing the control and ischemia-reperfusion groups, the *Cebpd*, but not the rest of the C/EBP family members, mRNAs were highly increased (Student’s *t* test; *P < 0.05, **P < 0.01, ***P < 0.001).

Transcriptional activation plays a critical and vital role during the initiation and sequential response of various acute and chronic inflammatory diseases. To explore the involvement of C/EBPs in renal transplantation and injury, the expression of C/EBPs was assessed using the GEO database GSE5104 (*n* = 12). In contrast to the *Cebpa* and *Cebpg* transcripts, the *Cebpd* transcript was significantly increased in the kidney isografts of the rats after kidney transplantation (Fig. 1A). Moreover, according to the GEO database GSE182793 (*n* = 12), among the various C/EBP family members, only *Cebpd* was upregulated in mouse kidneys after IRI (Fig. 1B). Although there are differences between the samples in the database and the experimental model that was used in this study, it is still implied that CEBPD is a significant and common effector in kidney IRI.

### Cebpd expression is increased in a U-IRI mouse model

**Fig. 2:**
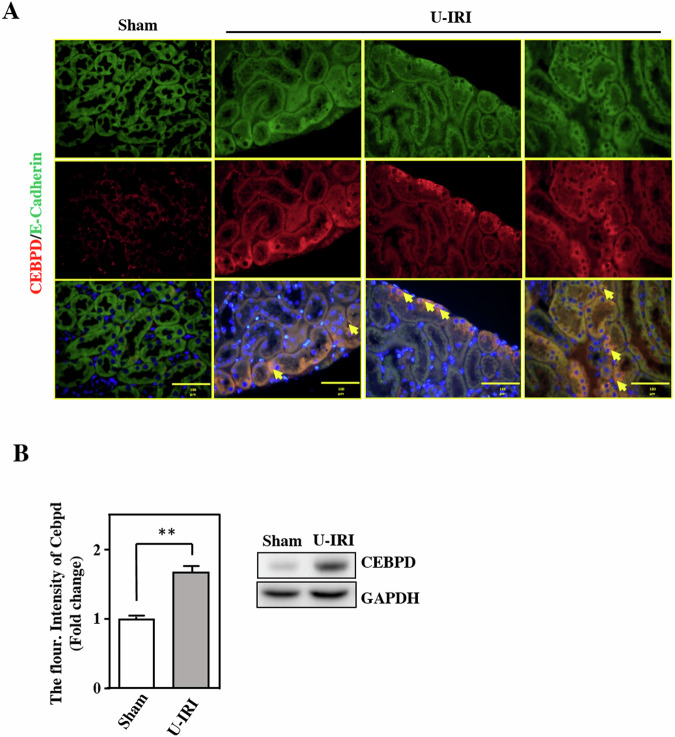
CEBPD expression is increased in a U-IRI mouse model. **A** Cebpd expression in U-IRI mice. Tubular epithelial cells were recognized with E-cadherin antibodies (green); CEBPD was recognized with specific CEBPD antibodies (red). The dead epithelial cells are marked with yellow arrows. Images were taken with a fluorescence microscope at a magnification of 200×. (*n* = 3 for each group). **B** Cebpd is mainly induced in the kidney epithelial cells of U-IRI mice following the quantification of fluorescence intensity using ImageJ software (Student’s *t* test; *P < 0.05, **P < 0.01, ***P < 0.001).

To address the involvement and contribution of CEBPD in renal injury, a U-IRI mouse model was established with a midline incision, thus allowing for ischemia-reperfusion of the left kidney and simultaneous right nephrectomy to mimic human kidney transplantation. In U-IRI mice, Cebpd signals were increased and overlapped with those of the epithelial cell marker E-cadherin, which indicated that Cebpd was primarily expressed in renal tubular epithelial cells, especially in necrotic epithelial cells (Fig. 2A, yellow arrow). Compared with other tubular epithelial injury features, including loss of the brush border (epithelial simplification), the expression of Cebpd was associated with the severity of necrosis (loss of nuclei) in epithelial cells. Moreover, the fluorescence intensity of Cebpd was quantified and the results demonstrated that the expression of Cebpd was increased in U-IRI mice compared with sham mice (Fig. 2B). In addition to immunofluorescence, immunohistochemical staining also showed that the expression of Cebpd was increased in U-IRI mice (Fig. 2A & Supplementary Fig. 1). These results imply that epithelial Cebpd plays a potential role in renal IRI.

### *Cebpd*-deficient mice exhibit attenuated U-IRI with reduced inflammatory cytokine release

**Fig. 3:**
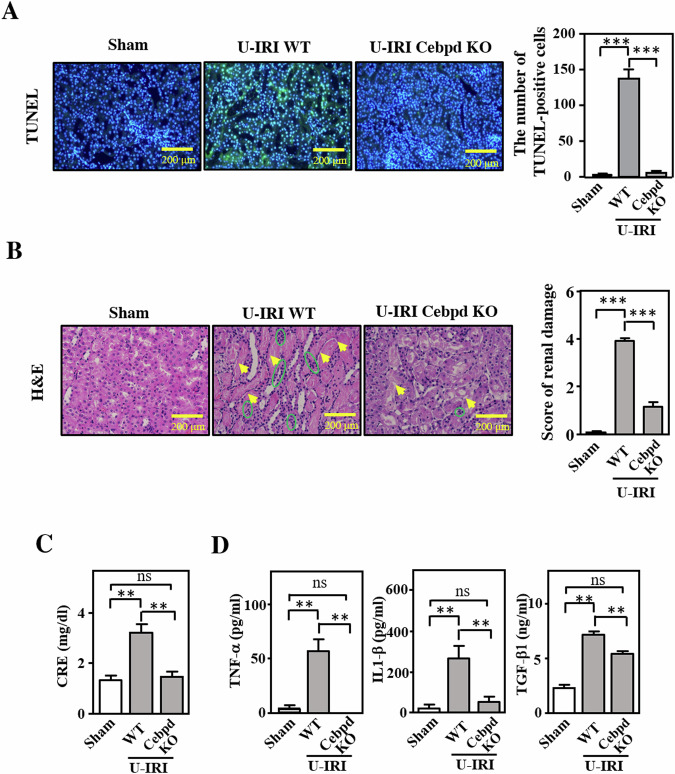
*Cebpd*-deficient mice exhibit attenuated IRI with reduced inflammatory cytokine release and FN-1 expression. **A** The apoptotic effect on the kidneys of U-IRI mice. A TUNEL assay was performed to measure the apoptosis of kidney cells. The images were taken using fluorescence microscopy. The number of apoptotic cells was measured by counting the number of double -positive TUNEL (green) and DAPI (blue) cells. (one-way ANOVA; *P < 0.05, **P < 0.01, ***P < 0.001). **B** Association between kidney injury and immune cell infiltration in U-IRI *Cebpd* wild-type (WT) and -deficient (Cebpd KO) mice. H&E staining was performed to demonstrate the cell morphology and types in the kidneys of U-IRI mice. The yellow arrow indicates acute tubular injury; the green circle shows the infiltrated immune cells. The renal damage score was quantified via H&E staining and renal histopathology. (one-way ANOVA; *P < 0.05, **P < 0.01, ***P < 0.001). **C** The loss of Cebpd attenuates the U-IRI-induced increase in creatinine levels. The creatinine levels in plasma samples harvested from U-IRI-treated *Cebpd*-WT and -deficient mice (*n* = 6 for each group) (one-way ANOVA; *P < 0.05, **P < 0.01, ***P < 0.001). **D** The levels of inflammatory indicators were attenuated in U-IRI-induced kidney injury. The levels of the inflammatory factors TNF-α, IL-1β and TGF-β1 in U-IRI-treated *Cebpd*-WT and -deficient mice were measured by ELISA (*n* = 6 for each group) (one-way ANOVA; *P < 0.05, **P < 0.01, ***P < 0.001).

To test and address the contribution of Cebpd to kidney ischemia-reperfusion injury, U-IRI was induced in *Cebpd*-deficient (*Cebpd*^−/−^) and wild-type (WT) mice. The apoptotic effect in the U-IRI kidney was examined using a TUNEL assay. The findings showed a reduction in apoptosis in *Cebpd*-deficient mice compared to wild-type mice (Fig. 3A). Hematoxylin and eosin (H&E) staining demonstrated that the regions of tissue injury (marked by the yellow arrow) and immune cell infiltration (marked by the green circle) were increased after U-IRI treatment (Fig. 3B). Moreover, as measured via H&E staining, renal damage was ameliorated in *Cebpd*-deficient mice (Fig. 3B). Moreover, creatinine (CRE) levels were significantly reduced in U-IRI-treated *Cebpd*-deficient mice (Fig. 3C). As mentioned above, CEBPD has been suggested to upregulate inflammation- and ECM production-related genes, and renal parenchymal cells have the potential to produce various types of proinflammatory cytokines in response to IRI. However, gene regulation in response to CEBPD in epithelial cells remains elusive. Triggered by IRI, many cytokines, including TNF-α, IL-1β and TGF-β1, have been suggested to participate in inflammatory responses [26]. The levels of TNF-α, IL-1β and TGF-β1 in mouse plasma were measured and the results showed that the levels of TNF-α, IL-1β and TGF-β1 were dramatically increased in U-IRI-treated mice but were significantly attenuated in U-IRI-treated *Cebpd*-deficient mice (Fig. 3D). These results suggest that inactivation of Cebpd/CEBPD can attenuate inflammatory responses in the kidney.

### The colocalization of the macrophage marker F4/80 with FN-1 is diminished in the kidneys of *Cebpd*-deficient mice following U-IRI

**Fig. 4:**
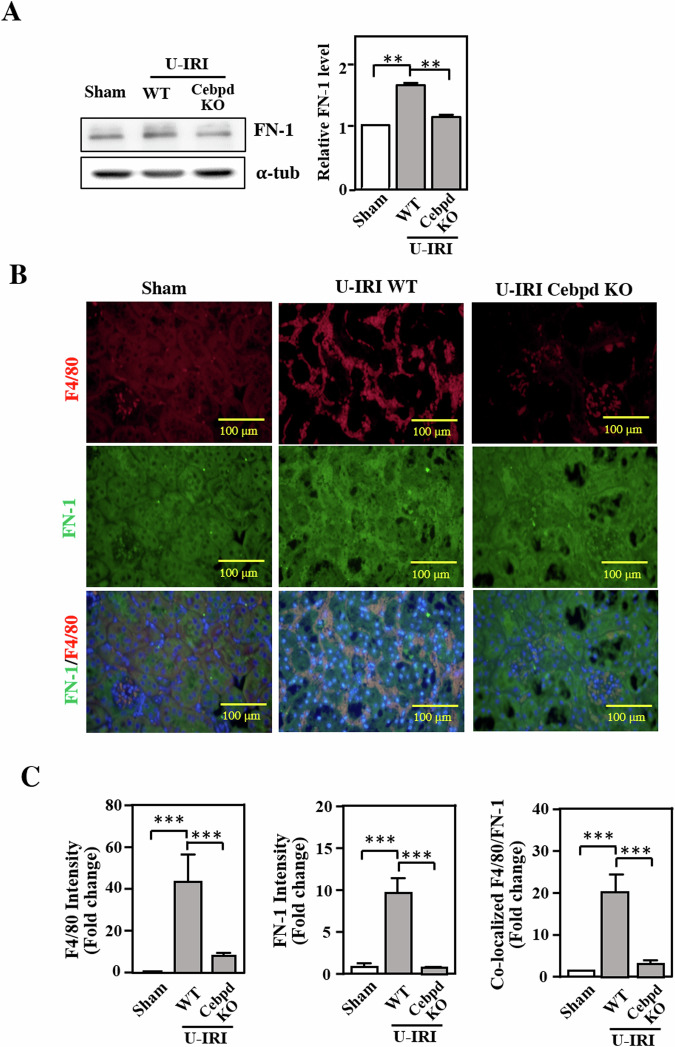
F4/80 and FN-1 colocalization was reduced in the kidneys of *Cebpd*-deficient mice following U-IRI. **A** FN-1 levels are attenuated in the kidneys of *Cebpd*-deficient mice following U-IRI administration. FN-1 expression was examined via Western blotting and quantified by statistical analysis. (one-way ANOVA; *P < 0.05, **P < 0.01, ***P < 0.001). **B** The number of macrophages surrounding epithelial cells was attenuated in the kidneys of U-IRI *Cebpd*-deficient mice. The macrophages were labeled with F4/80 (red) and FN-1 was detected using a specific anti-FN-1 antibody (green). The images were taken with a fluorescence microscope and magnified at 200×. **C** The fluorescence intensity of colocalized F4/80 and FN-1 signals in the kidneys of U-IRI mice was quantified by ImageJ software (*n* = 6 for each group) (one-way ANOVA; *P < 0.05, **P < 0.01, ***P < 0.001).

Our previous study showed that CEBPD contributes to ECM production in spinal cord injury and pancreatic cancer [27, 28]. FN-1 is an ECM component that induces and promotes macrophage migration and adhesion [13, 29]. To assess whether CEBPD contributes to FN-1 production in kidney epithelial cells, we also examined FN-1 expression in the kidneys of U-IRI mice. Compared with that in U-IRI-treated mice, the expression of FN-1 was significantly attenuated in U-IRI-treated *Cebpd*-deficient WT mice (Fig. 4A). As shown in Fig. 3B, reduced immune cell infiltration was observed in U-IRI-treated *Cebpd*-deficient mice. Moreover, an increase in F4/80-positive immune cells was observed in the kidneys of U-IRI-treated WT mice but decreased in those of U-IRI-treated *Cebpd*-deficient mice (Fig. 4B, C). Loss of macrophage accumulation surrounding the necrotic tubules in response to U-IRI in *Cebpd*-deficient mice suggests that Cebpd contributes to macrophage accumulation. To further verify whether Cebpd affects macrophage accumulation in renal IRI, an immunofluorescence assay was performed, and the results showed that, compared with that in U-IRI-treated WT mice, decreased FN-1 deposition in the kidneys of *Cebpd*-deficient mice was observed following U-IRI surgery (Fig. 4B, D). Importantly, colocalized F4/80 and FN-1 signals were mainly observed in renal tubular epithelial cells from U-IRI kidneys, thus suggesting that macrophage accumulation is associated with FN-1 expression in epithelial cells. In addition, neutrophil infiltration can be observed in renal IRI [30]. Our immunofluorescence staining demonstrated that neutrophil accumulation was also diminished in U-IRI-treated *Cebpd*-deficient mice, but the signals of the neutrophil marker Ly6G did not colocalize with epithelial FN-1 during renal injury (Supplementary Fig. 2). These results suggested that Cebpd-mediated FN-1 expression in epithelial cells specifically affects macrophage, but not neutrophil, accumulation.

### Loss of CEBPD through reduced fibronectin inhibits hypoxia-induced macrophage adhesion in HK-2 cells

**Fig. 5:**
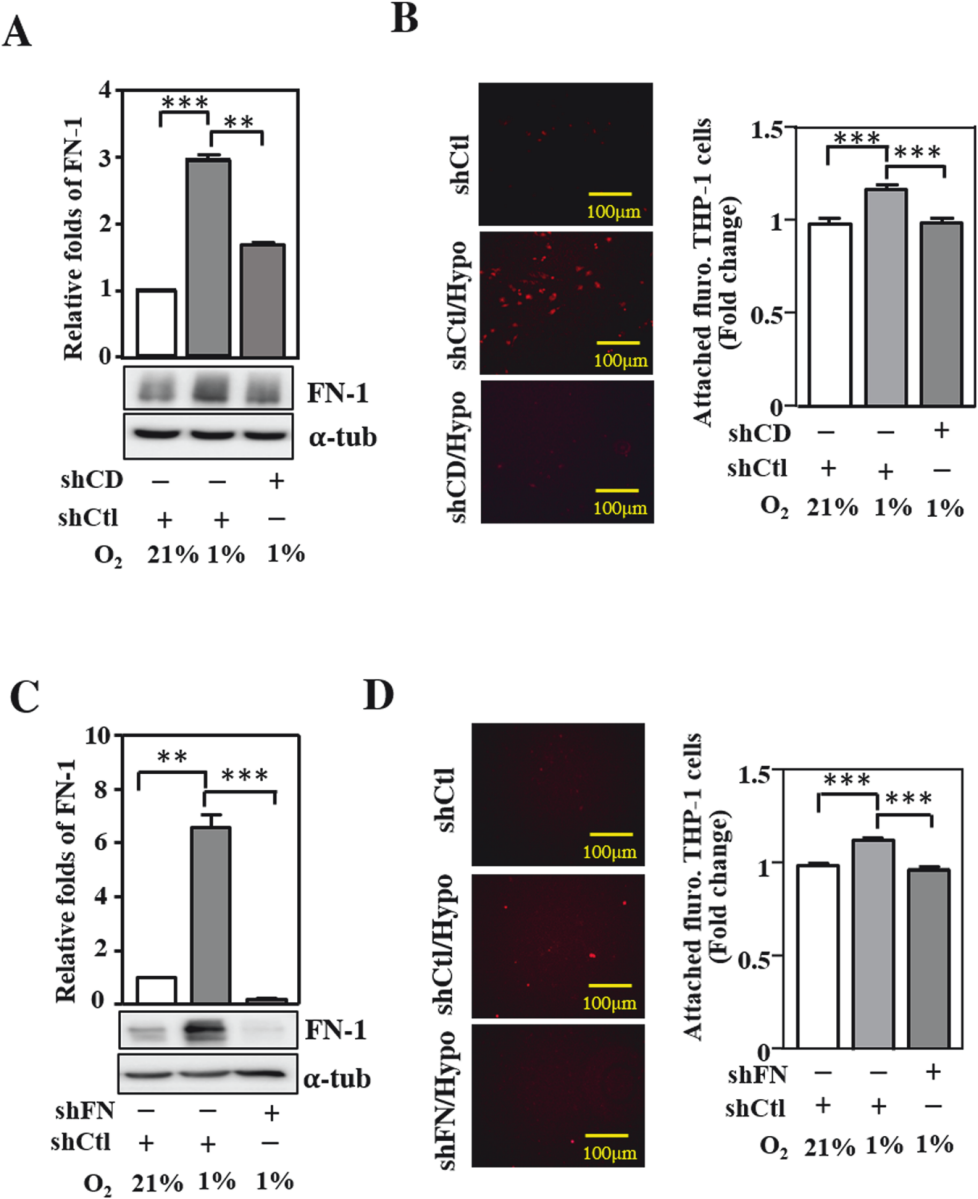
Loss of CEBPD attenuates hypoxia-induced macrophage adhesion to HK-2 cells. **A** FN-1 expression is attenuated in CEBPD knockdown HK-2 cells. FN-1 expression was analyzed in shControl (shCtl) and shCEBPD (shCD) HK-2 cells under normoxia (21% O_2_) or hypoxia (1% O_2_). (one-way ANOVA; *P < 0.05, **P < 0.01, ***P < 0.001). **B** Macrophage adhesion is attenuated in coculture with CEBPD knockdown HK-2 cells. mCherry THP-1 M1 macrophages were coincubated with shCtl or shCEBPD (shCD) HK-2 cells. After washing with PBS, an ELISA reader determined the number of attached fluorescent macrophages. (one-way ANOVA; *P < 0.05, **P < 0.01, ***P < 0.001). **C** FN-1 expression is attenuated in FN-1 knockdown HK-2 cells. FN-1 expression was analyzed in shControl (shCtl) and shCEBPD (shCD) HK-2 cells under normoxia (21% O_2_) or hypoxia (1% O_2_). (one-way ANOVA; *P < 0.05, **P < 0.01, ***P < 0.001). **D** Macrophage adhesion is attenuated in coculture with FN-1 knockdown HK-2 cells. mCherry THP-1 M1 macrophages were coincubated with shCtl or shFN-1 HK-2 cells. After washing with PBS, an ELISA reader determined the number of attached fluorescent macrophages. (one-way ANOVA; *P < 0.05, **P < 0.01, ***P < 0.001).

To verify whether CEBPD regulates FN-1 in renal epithelial cells, HK-2 cells (a human renal tubular epithelial cell line) were used and subjected to a loss-of-function assay. CEBPD and FN-1 expression was upregulated under hypoxic conditions in HK-2 cells, and the expression of both genes was attenuated in CEBPD knockdown HK-2 cells in response to hypoxia (Fig. 5A, B). In addition, a previous study demonstrated that, compared to M2 macrophages, M1 macrophages showed stronger adhesion activity [31]. Afterward, we evaluated whether macrophage adhesion was modulated by CEBPD activation in HK-2 cells. mCherry THP-1 M1 macrophages, differentiated by PMA, interferon-gamma and lipopolysaccharide [31], were coincubated with parental and CEBPD knockdown HK-2 cells to assess adhesion under hypoxic conditions. Compared to coincubation with parental HK-2 cells, the adhesion activity of THP-1 M1 macrophages was attenuated in CEBPD knockdown HK-2 cells under hypoxic conditions (Fig. 5C). Moreover, following the confirmation of FN-1 knockdown in HK-2 cells (Fig. 5D), the contribution of FN-1 to macrophage adhesion was assessed. The results showed that after coincubation of THP-1 M1 macrophages with CEBPD knockdown HK-2 cells, the adhesion of macrophages was attenuated in FN-1 knockdown HK-2 cells under hypoxic conditions (Fig. 5G). These results suggest that epithelial CEBPD and FN-1 contribute to macrophage adhesion.

**Fig. 6:**
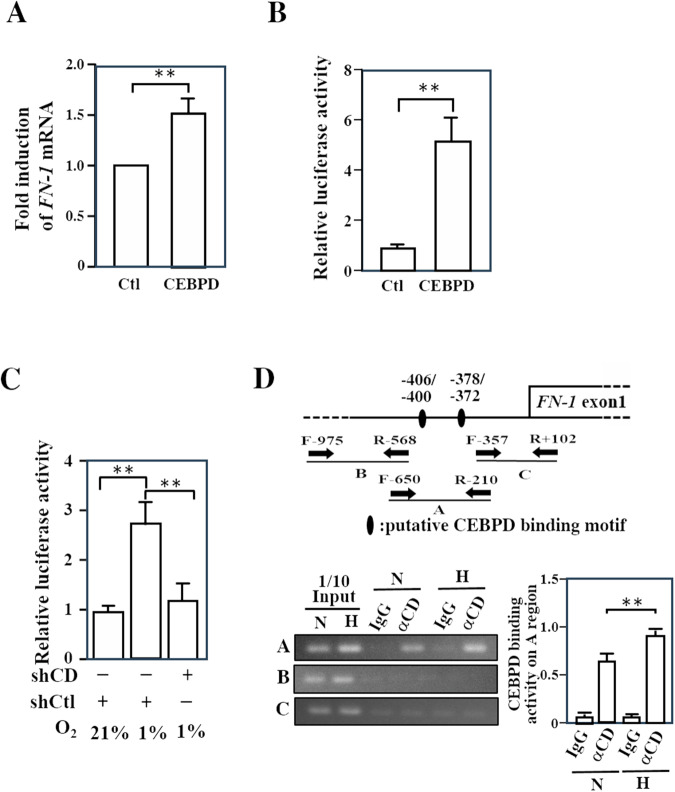
CEBPD directly contributes to *FN-1* gene activation in HK-2 cells. **A** Exogenous CEBPD activates *FN-1* transcription in HK-2 cells. A RT‒qPCR assay was performed with total RNA from control and CEBPD-expressing vector transfectants. (Student’s *t* test; *P < 0.05, **P < 0.01, ***P < 0.001). **B** Exogenous CEBPD activates the *FN-1* reporter in HK-2 cells. A reporter assay was performed with cell lysates from the *FN-1* reporter and control or CEBPD-expressing vector transfectants. (Student’s *t* test; *P < 0.05, **P < 0.01, ***P < 0.001). **C** The inactivation of CEBPD attenuates hypoxia-induced *FN-1* reporter activity in HK-2 cells. A reporter assay was performed with cell lysates from *FN-1* reporter and lentiviral shcontrol (shCtl)- or shCEBPD (shCD)-infected transfectants. (one-way ANOVA; *P < 0.05, **P < 0.01, ***P < 0.001). **D** CEBPD (CD) directly binds to the *FN-1* gene promoter and this binding is responsive to hypoxia in HK-2 cells. A ChIP-PCR assay was performed with the indicated antibody-pull down formaldehyde-treated HK-2 cell lysates under normoxic (N) or hypoxic (H) conditions (Student’s *t* test; *P < 0.05, **P < 0.01, ***P < 0.001).

As mentioned above, CEBPD is a transcription factor and FN-1 contributes to an increase in the adhesion of various cells [32–34]. We further tested whether FN-1 is a downstream target of CEBPD in kidney epithelial cells. Firstly, co-transfection of the *FN-1* reporter and the CEBPD expression vector in HK-2 cells was performed to test whether *FN-1* transcription and reporter activity are responsive to CEBPD activation in epithelial HK-2 cells. The results demonstrated that CEBPD can activate *FN-1* transcription and reporter activity in HK-2 cells (Fig. 6A, B). We subsequently tested whether hypoxia could activate *FN-1* reporter activity and whether the inactivation of CEBPD could disrupt hypoxia-induced *FN-1* reporter activity. The results showed that *FN-1* reporter activity was activated under hypoxic conditions and that the inactivation of CEBPD attenuated hypoxia-induced *FN-1* reporter activity (Fig. 6C). Moreover, a ChIP assay was conducted to determine whether CEBPD could directly bind to the *FN-1* promoter region in HK-2 cells. Two putative CEBPD binding motifs on the *FN-1* promoter were predicted using PROMO 3.0 (https://alggen.lsi.upc.es/cgi-bin/promo_v3/promo/promoinit.cgi?dirDB=TF_8.3; Fig. 6D upper panel). The results of ChIP assay showed that the binding of CEBPD was increased in HK-2 cells under hypoxic conditions (Fig. 6D, lower panel). These results suggested that CEBPD can directly bind to the *FN-1* promoter and that CBEPD binding is responsive to hypoxia in HK-2 cells.

## Discussion

**Fig. 7:**
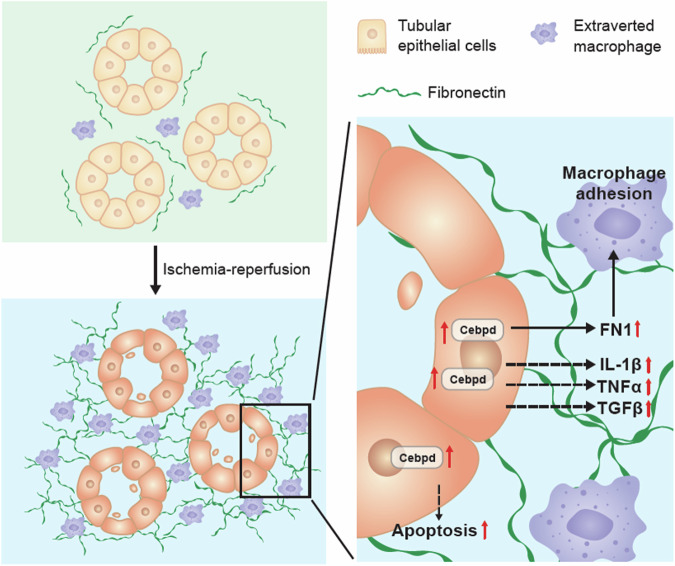
A schematic diagram illustrates the proposed model. In response to IRI, CEBPD can be activated in epithelial cells. CEBPD activation is associated with apoptosis and contributes to the transcriptional activation of the *FN-1* gene in epithelial cells. An increase in FN-1 can enhance macrophage adhesion and is involved in inflammatory activation and kidney injury.

IRI in renal transplantation is closely linked to a myriad of complications, including delayed graft function, graft rejection, and chronic graft dysfunction, which is due to its impact on diverse cellular regulatory systems that induce a distinct inflammatory response in the transplanted kidney. This finding underscores the significance of IRI (which is believed to be the foremost nonspecific factor influencing both early and late allograft dysfunction) as a critical concern in kidney transplantation [35–38]. To improve renal graft outcomes, the underlying mechanisms of IRI to the graft are very important for defining strategies to prevent or treat IRI after kidney transplantation. In this study, we found that CEBPD can directly bind to the promoter of the *FN-1* gene and consequently contribute to the enhancement of macrophage attachment, which is involved in the pathogenesis of renal IR (Fig. 7).

The determination of the pathophysiology of renal IRI via a good experimental model that reproducibly induces acute kidney injury (AKI) is critical. Many investigators have utilized bilateral renal pedicle clamping as a renal IRI model to examine the molecular pathophysiology of AKI. To mimic IRI after single kidney transplantation, the contralateral nephrectomy was replaced by a unilateral IRI model [39]. In this U-IRI mouse model, renal damage and elevated creatinine were observed. Moreover, the decline in renal function was inhibited by *Cebpd* deficiency (Fig. 3B, D).

In previous studies, CEBPD was shown to be upregulated by many extracellular stimuli, such as IL-1β, IL-6, TNF-α, IFN-α, IFN-γ, LPS and TGF-β1 [27, 40–42]. In hypoxia, the activation of CEBPD has been suggested to be associated with HIF-1α activity and cellular function in profibrotic and protumor conditions [25, 43–45]. However, the associations between CEBPD and macrophage attachment and renal fibrosis remain poorly characterized. CEBPD is also reciprocally implicated in the activation of TNF-α, IL-1β and IL-6 [46]. In addition, monocytes/macrophages that appear in kidney injury produce inflammation-associated cytokines, such as IL-1, IL-6 and TGF-β [47, 48]. Consistent with these results, the levels of TNF-α, IL-1β and TGF-β1 in the plasma and the level of macrophage recruitment to the kidney were reduced in *Cebpd*-deficient mice after U-IRI (Figs. 3B, D and 4B). These findings suggest that reduced disease susceptibility in *Cebpd*-deficient mice is associated with a reduced inflammatory response. As Cebpd influences the regulation of cytokine-producing inflammatory cells, epithelial Cebpd-mediated inflammatory cell recruitment is expected to play an important role in renal IRI. However, a recent study suggested that TNF-α, IL-1β and TGF-β are also expressed by renal epithelial cells after renal injury [49]. Accordingly, the interactions among renal tubular epithelial cells, inflammatory cells and cytokines in acute kidney injury require further investigation.

Typically, the damaged tubular epithelium releases proinflammatory cytokines and chemokines, thus triggering and intensifying inflammatory responses. Subsequently, inflammatory cells are recruited and cause direct injury to tubular epithelial cells. However, some leukocytes migrate through the endothelium into the interstitial compartment [20]. Additionally, ECM proteins are thought to be endogenous danger signals in inflamed tissue and can stimulate resident immune cells in the absence of microbial infection [50]. I In the current study, we demonstrated that Cebpd was primarily expressed in renal tubular epithelial cells, especially in the necrotic epithelium, and regulated the expression of FN-1 (which is an ECM component) following U-IRI. Furthermore, the colocalization of the macrophage marker F4/80 with FN-1 in renal injuries showed that the overlap was mainly localized in the cytoplasm of renal tubular epithelial cells (Fig. 4). Altered expression of FN-1 is observed in some pathological situations, including inflammation [51, 52]. Moreover, excessive macrophage accumulation can exacerbate inflammatory diseases [20]. Collectively, in renal tubular epithelial cells, CEBPD-induced FN-1 can mediate macrophage accumulation directly to the site of tubular injury and be conducive to renal IRI.

Tissue injury elicits innate immune responses followed by the local production of chemokines, which recruit neutrophils, naïve monocytes and macrophages to sites of inflammation [53]. Both neutrophils and macrophages are effective at eliminating pathogens; however, neutrophils and macrophages found in IRI are likely to extend the early injury phase of tubular epithelial cells. In the present study, CEBPD activation mediated kidney injury after IRI through macrophage and neutrophil accumulation, but the signals of the neutrophil marker Ly6G did not colocalize with FN-1 in renal injury (Supplementary Fig. 2). Therefore, the molecular mechanisms underlying CEBPD-enhanced neutrophil accumulation require further analysis. In injured kidneys, inflammation is induced by resident tubular cells and inflammatory cells, particularly macrophages [42]. Monocytes and macrophages infiltrate tissue within a few days after IRI; however, the direct role of macrophages in mediating early tubular injury is still being investigated. Macrophage infiltration appears in the mouse kidney within 1 h after reperfusion; it then peaks at 24 h and persists for 7 days [20].

Depletion of macrophages prior to IRI was shown to attenuate renal injury in a murine IRI model [54]. A previous study showed that blockade or deficiency of C-C motif chemokine receptor 1, which is an immune cell accumulation regulator, leads to decreased macrophage accumulation in the damaged kidney but cannot alter the degree of renal injury [55]. In this study, we demonstrated that epithelial CEBPD contributes to macrophage accumulation by activating FN-1-mediated cell adhesion (Fig. 5B, D). A previous study demonstrated that under hypoxic conditions, CEBPD can bind to the *FN-1* promoter and activate FN-1 expression in cancer cells [25]. Our current results agree with this observation, thus indicating that CEBPD could be a common activator of *FN-1* transcriptional activation under hypoxic conditions. In addition, FN-1 deamidation on the Asn-Gly-Arg region can promote monocyte adhesion [56]. Therefore, whether CEBPD can potentially affect the deamination of FN-1 and its consequent effect on FN-1 stability can be tested in the future.

## Materials and methods

### A uninephrectomized mouse model of ischemia-reperfusion injury (U-IRI)

Twelve- to 14-week-old male C57BL/6J mice were acquired from BioLASCO Taiwan Co. *Cebpd*^*−/−*^ mice (on a C57BL/6 background) were generously provided by Sterneck [57], and reproduction was performed at the National Laboratory Animal Center. The abdomens of the male C57BL/6 mice were opened under mixed anesthesia. The left renal pedicle was occluded for 45 min with a microvascular clip, followed by 24 h of reperfusion. During the ischemic period, dissection was continued to release the right kidney’s lateral and posterior renal attachments. The right kidney was retracted inferiorly to permit the remaining upper pole attachment dissection. The right renal hilum was isolated and ligated with 4-O silk, and the right kidney was carefully removed. Adequate restoration of the left renal blood flow was checked before abdominal closure. Before wound closure, warm sterile saline was intraperitoneally administered to each mouse. The experimental mice were sacrificed, and the remaining kidney was removed after 24 h of reperfusion. Kidney tissues and blood samples were taken for analysis. The remnant kidney was harvested and transversally cut at the midline. One-half of the kidneys were fixed in formalin and embedded in paraffin. The other sample was homogenized, and the protein extraction solution was added for Western blotting. Animal procedures followed protocols approved by the Institutional Animal Care and Use Committee of National Cheng Kung University (approval IACUC number: 109121).

### Cell culture

The human proximal tubular cell line HK-2 cells were cultured in Dulbecco’s Modified Eagle Medium/Nutrient Mixture F-12 (Thermo Fisher Scientific Inc., USA #11320033) supplemented with 10% fetal calf serum. The human monocyte cell line THP-1 cells were maintained in RPMI 1640 Medium (Thermo Fisher Scientific Inc., USA #11875119) supplemented with 10% fetal calf serum.

### Cell hypoxia

Cell hypoxia (0.1% O_2_) was induced with an anaerobic bag (Mitsubishi Gas Chemical Company, Inc., Japan). The experimental HK-2 cells were incubated in 0.1% O_2_ at 37 °C for 24 h, and phorbol 12-myristate 13-acetate (PMA) (320 nM)/IFN-gamma (20 ng)/LPS (100 ng)-differentiated THP-1 cells were then incubated with HK-2 cells for 10 min. After coculture, the experimental cells were gently washed with PBS to remove the unattached cells. Subsequently, all of the cells were collected in trypsin-EDTA buffer, and multimode microplate readers were used to detect the number of fluorescent THP-1 cells.

### Renal histopathology

Renal tissue samples were immersed in 10% formalin for 24 h for fixation and subsequently embedded in paraffin. Histological analysis was performed with hematoxylin staining. The severity of renal damage was scored on hematoxylin and eosin (H&E)-stained sections by grading the percentage of tubule injury that showed loss of the proximal tubule brush border, cell swelling or vacuolization, and cell necrosis as follows: 0 (<1%); 1 (1–10%); 2 (11–20%); 3 (21–40%); 4 (41–60%); 5 (61–75%); and 6 (>75%) [58]. The score ranges of 1–2 represent mild injury, 3–4 represent moderate injury, and 5–6 represent severe injury. Ten random fields of view from the corticomedullary junction, known as the most susceptible area of the kidney to ischemia-reperfusion injury, were observed on each slide section at a magnification of 200×.

### Western blot analysis

The tissues were dissected and frozen on dry ice. Tissues or experimental cells were homogenized in lysis buffer (0.05 M Tris-HCl, 0.15 M NaCl, 1 mM EDTA, 1%NP40, 0.25% NaDOC, 1 μg/ml Aprotinin, 1 μg/ml Leupeptin, 100 μg/ml PMSF, 1 mM DTT, 1 mM Na_3_VO_4_, 0.5 μM NaF and 0.01% SDS). Sections were incubated with primary antibodies, including anti-CEBPD (GTX115047; GeneTex), anti-GAPDH (GTX100118; GeneTex), anti-FN-1(#15613-1-AP; Proteintech) and anti-α-tubulin (T6199; Sigma), and the signals were visualized using an enhanced chemiluminescence Western blot system.

### Immunofluorescence analysis

Tissue sections were cut from frozen kidney blocks onto precoated slides. The slides were subjected to a 1-hour treatment with a protein blocker. Antigen retrieval was performed by heating the slides to 121 °C in 10 mM citrate buffer (pH 6) for 20 min, followed by washing with phosphate-buffered saline. Subsequently, the slides were incubated with specific antibodies targeting CEBPD (sc‐365546; Santa Cruz Biotechnology), F4/80 (sc-377009; Santa Cruz Biotechnology), FN-1 (#15613-1-AP; Proteintech), and E-cadherin (GTX100443; GeneTex), each antibody was diluted at a ratio of 1:200. The slides were treated with secondary antibodies conjugated with Alexa 488 and 594 (diluted at a ratio of 1:200) and then incubated for 1 h at room temperature. The images were observed with an Olympus fluorescence microscope (BX51) and quantified via densitometry with ImageJ. The quantification method involved capturing images of 10 randomly selected regions from each slide and quantifying them using ImageJ. Statistical analysis was performed by comparing the values of other groups with the mean value of the Sham group as the reference.

### ELISA of chemokines in plasma samples

Concentrations of the chemokines IL-1β, TNF-α and TGF-β1 were measured in mouse plasma samples using R&D ELISA kits according to the manufacturer’s protocol (IL-1β #DY401-05, TNF-α #DY410-05, TGF-β1 #DY1679-05). For renal injury, the creatinine (CRE) concentration was measured from the plasma samples of sacrificed experimental mice after one day of reperfusion.

### Short hairpin RNA (shRNA) assay

Lentiviruses were generated by transfecting Phoenix cells with the indicated shRNA expression vectors and pMD2.G and psPAX2 plasmids. After viral infection efficiency was confirmed, HK-2 and THP-1 cells were infected with shCEBPD and mCherry lentiviruses, respectively, for 48 h. The lentiviral expression vectors were purchased from the National RNAi Core Facility at the Genomic Research Center of the Institute of Molecular Biology, Academia Sinica, Taiwan. The sequences of the indicated target genes were as follows: shCDBPD, 5′-CCGGGCTGTCGGCTGAGAACGAGAACTCGAGTTCTCGTTCTCAGCCGACAGCTTTTT-3′; shControl, 5′-CCGGAGTTCAGTTACGATATCATGTCTCGAGACATTCGCGAGTAACTGAAC TTTTTT -3′.

### Apoptosis assay

Cell apoptosis was analyzed according to the instructions of the terminal deoxynucleotidyl transferase dUTP nick end labeling (TUNEL) assay kit (# E-CK-A331, Elabscience company, USA). Fluorescence images were taken by fluorescence microscopy (BX51, Olympus). Capture ten images at 100X magnification and count the number of apoptotic cells in each image.

### Reporter plasmids and luciferase assay

The promoter region of the *FN-1* gene was obtained from the genomic DNA of HK-2 cells via PCR with the following specific primers: forward, 5′-CGACGCGTCGCGTGGGAAAGGACACGAAGA-3′ and reverse 5′-CCGCTCGAGCGGGCCACCAAGTTTGCTTCCCTTC-3′. The amplified DNA from the −976~+233 region of the *FN1* gene locus was further subcloned and inserted into the pGL3-basic vector. For the luciferase assay, the *FN-1* reporter vector was cotransfected with pCDNA3 with or without HA/CEBPD cDNA in HK-2 cells or transfected into HK-2 cells infected with lentivirus containing shCEBPD or shControl sequences using the transfection reagent TransIT-2020 (Mirus) for 18 h, or it was further incubated under hypoxic or normoxic conditions for another 24 h.

### Chromatin immunoprecipitation assay

Chromatin immunoprecipitation (ChIP) was performed as described by Wang et al. [59] Briefly, following exposure to normoxia (21% O_2_) or hypoxia (1% O_2_), various treatments were used. Experimental HK-2 cells, including those treated with lentivirus containing shCtl or shCEBPD (shCD), were fixed with 1% formaldehyde. The chromatin was cross-linked and subsequently prepared and sonicated to an average size of 500 bp. The DNA fragments were subjected to immunoprecipitation overnight at 4 °C using antibodies specific for CEBPD or control rabbit immunoglobulin G (IgG). The immunoprecipitated chromatin was amplified using primers targeting specific regions after cross-linking reversal. The sequences of the paired primers are as follows: region A (5′-AAGAAGTCCGAACAGGGAGC-3′ and 5′-AAAGAGATGCTGATGGCCCG-3′), region B (5′-CGTGGGAAAGGACACGAAGA-3′ and 5′-ATCCCGCTCCCTTTCTTTGG-3′) and region C (5′-CCCCTTCGCTTCACACAAGT-3′ and 5′-AAGGGATGCAGAGGACCAGA-3′). After PCR, the amplified products were analyzed via agarose gel electrophoresis.

### Statistical analysis

All experiments were performed with a minimum of three independent replicates. The statistical analysis was performed using a two-tailed unpaired Student’s *t* test and one-way ANOVA, with a significance threshold set at *p* < 0.05. The statistical analysis was conducted using GraphPad Prism software. The values plotted are depicted as the means with standard error of the means (SEMs).

### Supplementary information

Supplemental Figure

Western blot original data

## Data Availability

All data associated with this study are presented in the paper.
