# Supplementary material for: Epithelial CEBPD activates fibronectin and enhances macrophage adhesion in renal ischemia-reperfusion injury
Source: Cell Death Discov. 2024 Jul 18;10:328. doi: 10.1038/s41420-024-02082-4 (PMC11258324; doi:10.1038/s41420-024-02082-4)
Supplement: Supplementary file 1 — Supplemental Figure [file 41420_2024_2082_MOESM1_ESM.pdf]

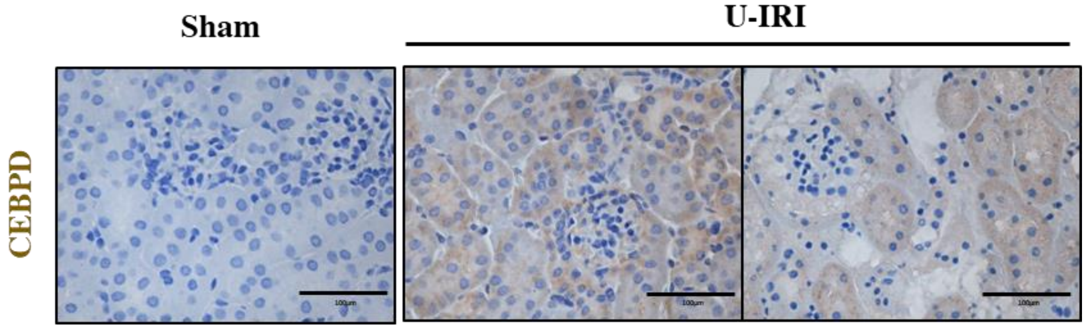

Supplementary Fig.1 Cebpd is activated in U-IRI mice.

The level of Cebpd was detected in paraffin-embedded kidney sections from U-IRI mice via immunohistochemistry.

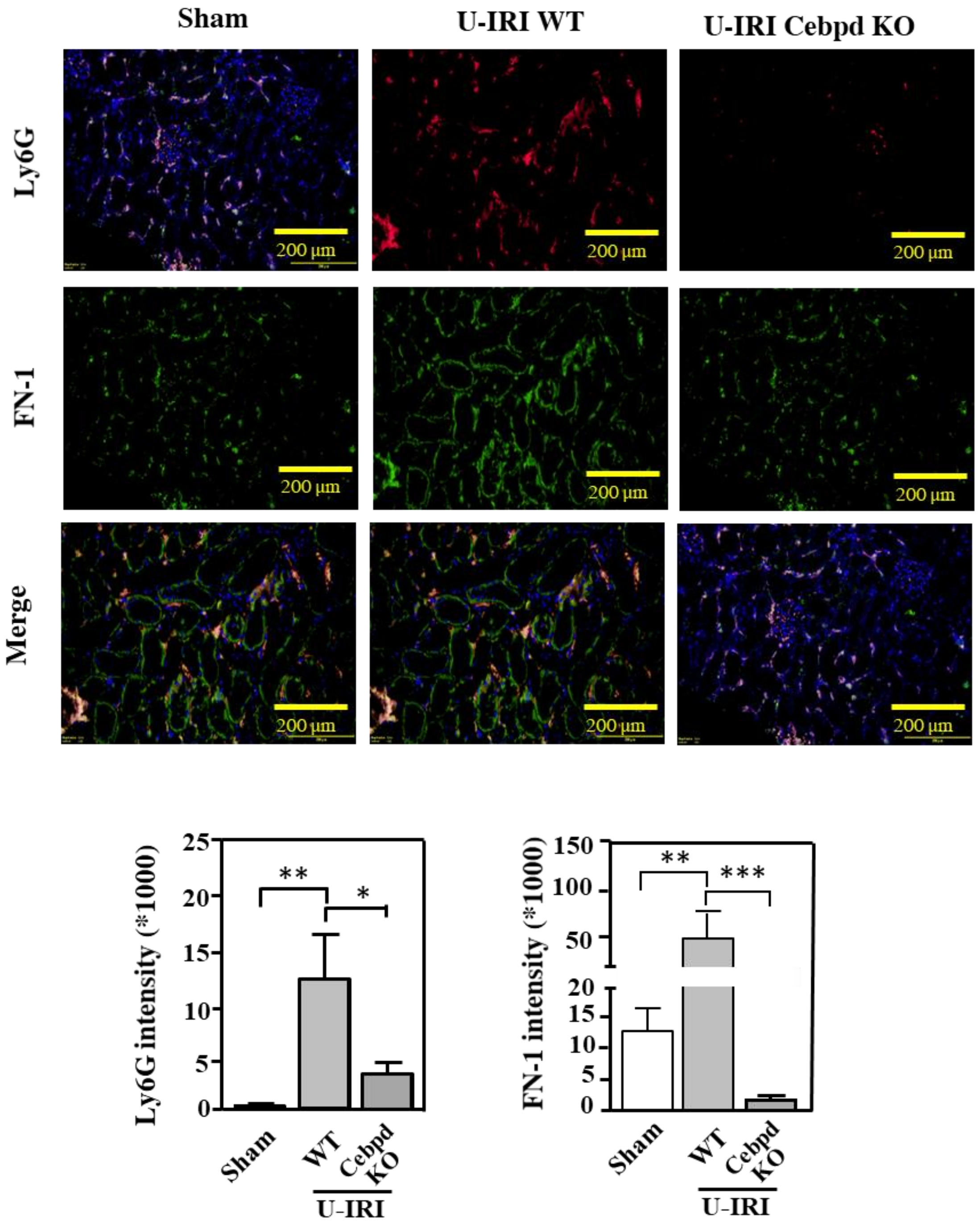

Supplementary Fig. 2 FN-1 signals are distinguished from Ly6G^{+} signals in the kidneys of U-IRI mice.

The representation of FN-1 and Ly6G^{+} signals in renal U-IRI. Neutrophil cells labeled with Ly6G (red) and FN-1 were identified using a specific anti-FN-1 antibody and are labeled in green. The

images were taken with a fluorescence microscope and magnified at 200x. The fluorescence intensities of the Ly6G and FN-1 signals in the kidneys of U-IRI mice were quantified by ImageJ software (one-way ANOVA; *=$P<0.05$, **=$P<0.01$, ***=$P<0.001$)

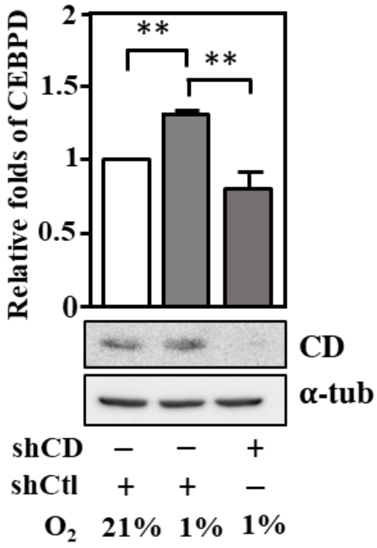

Supplementary Fig. 3 CEBPD expression is attenuated in CEBPD knockdown HK-2 cells.

CEBPD expression was analyzed in shControl (shCtl) and shCEBPD (shCD) HK-2 cells under normoxia (21% O_2) or hypoxia (1% O_2). (one-way ANOVA; *=P<0.05, **=P<0.01, ***=P<0.001)
