# Supplementary figures and images for: Epithelial CEBPD activates fibronectin and enhances macrophage adhesion in renal ischemia-reperfusion injury
Source: Cell Death Discov. 2024 Jul 18;10:328. doi: 10.1038/s41420-024-02082-4 (PMC11258324; doi:10.1038/s41420-024-02082-4)

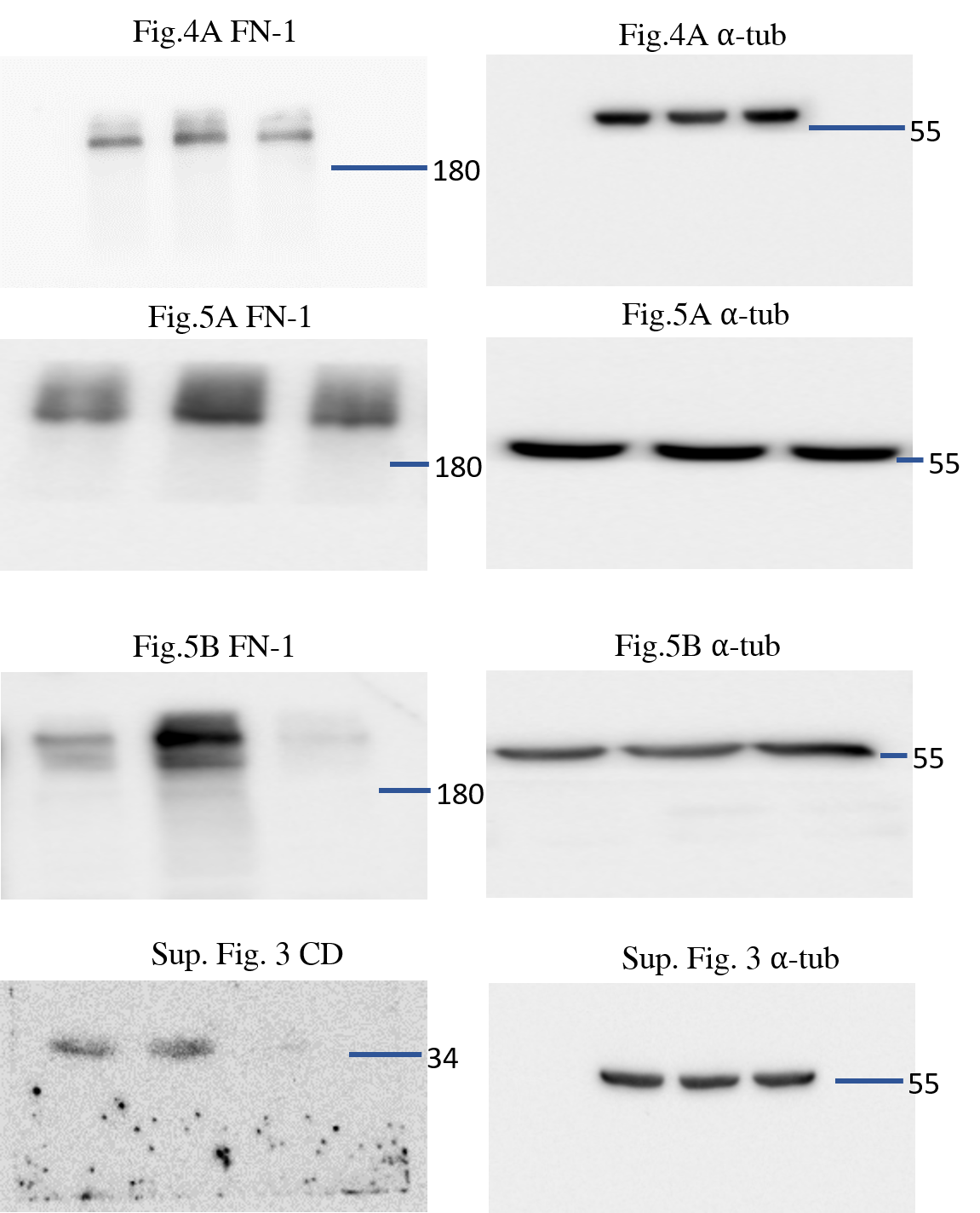

Supplement: Supplementary file 2 — Western blot original data [file 41420_2024_2082_MOESM2_ESM.tif]
